# Supplementary material for: Sleep Health Promotion Interventions and Their Effectiveness: An Umbrella Review
Source: Int J Environ Res Public Health. 2021 May 21;18(11):5533. doi: 10.3390/ijerph18115533 (PMC8196727; doi:10.3390/ijerph18115533)
Supplement: Supplementary file 1 [file ijerph-18-05533-s001.zip › ijerph-1214589-supplementary.pdf]

Table S1. Ratings of the included reviews using the JBI Quality Assessment Criteria for Systematic Reviews

	**Assessed items**										
**Reference**	**Is the review question clearly and explicitly stated?**	**Were the inclusion criteria appropriate for the review question?**	**Was the search strategy appropriate?**	**Were the sources and resources used to search for studies adequate?**	**Were the criteria for appraising studies appropriate?**	**Was critical appraisal conducted by two or more reviewers independently?**	**Were the methods used to combine studies appropriate?**	**Was the likelihood of publication bias assessed?**	**Were recommendations for policy and/or practice supported by the reported data?**	**Were the specific directives for new research appropriate?**	**Total**
Arora & Taheri (2017) [35]	0	1	1	1	0	0	1	0	0	1	5
Aslund et al. (2018) [36]	1	1	1	1	1	1	1	0	0	1	8
Barger et al. (2018) [41]	1	1	1	1	1	1	1	0	1	1	9
Blunden et al. (2012) [37]	1	1	1	1	1	1	1	0	1	1	9
Bonnar et al. (2018) [42]	1	1	1	1	1	1	1	0	1	1	9
Bryanton et al. (2013) [29]	1	1	1	1	1	1	1	1	1	1	10
Busch et al. (2017) [38]	1	1	1	1	1	1	1	0	1	1	9
Chung et al. (2017) [39]	1	1	1	1	1	1	1	1	1	1	10
Chung et al. (2017) [43]	1	1	1	1	1	1	1	1	1	1	10
Crichton & Symon (2016) [32]	1	1	1	1	0	0	1	0	1	1	7
De Niet et al. (2009) [52]	1	1	1	1	1	1	1	1	1	1	10
Dietrich et al. (2016) [30]	1	1	1	1	1	1	1	0	1	1	9
Du et al. (2015) [54]	1	1	1	1	1	1	1	1	1	0	9
Friedrich & Schlarb (2017) [44]	1	1	1	1	1	0	1	1	1	0	8
Halal & Nunes (2014) [47]	1	0	1	1	0	0	0	0	1	0	4

Hellström et al. (2011) [50]	1	1	1	1	1	1	1	0	1	0	8
Hollenbach et al. (2013) [51]	1	1	1	1	1	1	1	0	1	1	9
Hwang & Shin (2015) [58]	1	0	1	1	1	1	1	1	1	1	9
Kempler et al. (2015) [33]	1	1	1	1	1	1	1	1	1	1	10
Knowlden et al. (2016) [63]	1	1	1	1	1	0	1	0	1	1	8
Koch et al. (2006) [56]	0	1	1	1	0	1	0	0	0	1	5
Marx et al. (2017) [61]	1	1	1	1	1	1	1	1	1	1	10
Meltzer et al. (2014) [40]	1	1	1	1	1	1	1	1	1	1	10
Mihelic et al. (2017) [62]	1	1	1	1	1	0	1	0	1	1	8
Mindell et al. (2006) [34]	1	1	1	1	0	1	0	0	1	1	7
Minges & Redeker (2016) [60]	1	1	1	1	0	0	1	0	1	1	7
Morgenthaler et al. (2016) [59]	1	1	1	1	0	0	1	0	1	1	7
Murawski et al. (2018) [45]	1	1	1	1	1	0	1	0	1	1	8
Neuendorf et al. (2015) [31]	1	1	0	1	1	1	0	0	1	1	7
Owais et al. (2018) [46]	1	1	1	1	1	1	1	1	1	1	10
Rubio et al. (2017) [53]	1	1	1	1	1	1	1	1	1	1	10
Slanger et al. (2016) [49]	1	1	1	1	1	1	1	1	1	1	10
Tamrat et al. (2013) [48]	1	1	1	1	1	1	1	1	1	1	10
Wu et al. (2015) [57]	1	1	1	1	1	1	1	1	1	1	10
Yang et al. (2012) [55]	1	1	1	1	1	1	1	0	1	1	9
